# Cardiopulmonary resuscitation; use, training and self-confidence in skills. A self-report study among hospital personnel

**DOI:** 10.1186/1757-7241-16-18

**Published:** 2008-12-16

**Authors:** Laila A Hopstock

**Affiliations:** 1 Institute of Community Medicine, Faculty of Medicine, University of Tromsø, N-9037 Tromsø, Norway

## Abstract

**Background:**

Immediate start of basic cardiopulmonary resuscitation (CPR) and early defibrillation have been highlighted as crucial for survival from cardiac arrest, but despite new knowledge, new technology and massive personnel training the survival rates from in-hospital cardiac arrest are still low. National guidelines recommend regular intervals of CPR training to make all hospital personnel able to perform basic CPR till advanced care is available. This study investigates CPR training, resuscitation experience and self-confidence in skills among hospital personnel outside critical care areas.

**Methods:**

A cross-sectional study was performed at three Norwegian hospitals. Data on CPR training and CPR use were collected by self-reports from 361 hospital personnel.

**Results:**

A total of 89% reported training in CPR, but only 11% had updated their skills in accordance with the time interval recommended by national guidelines. Real resuscitation experience was reported by one third of the respondents. Both training intervals and use of skills in resuscitation situations differed among the professions. Self-reported confidence decreased only after more than two years since last CPR training.

**Conclusion:**

There is a gap between recommendations and reality in CPR training among hospital personnel working outside critical care areas.

## Background

The performance of cardiopulmonary resuscitation (CPR) has an important position in the chain of survival, but despite new techniques and technology the survival rates from cardiac arrest are still low. For in-hospital cardiac arrests, the overall survival rate is estimated to be less than 20% [1,2] and even lower outside critical care areas [3]. Recent research has focused on the quality of basic CPR skills and the use of automated external defibrillators (AEDs) by first responders. Several studies have shown that hospital personnel perform ineffective CPR, possibly due to irregular training and low skill retention [1,4]. Since 2003 the Norwegian national guidelines have recommended regular CPR training minimum every sixth month and CPR update courses once a year. The 2008 update of resuscitation guidelines from the Norwegian Resuscitation Council recommends CPR update course every second year and training every six months [5]. CPR training programs are continuously offered to all staff at most hospitals. The aim of this study is to assess how much CPR training hospital personnel working outside critical care areas have, what they have experienced from real resuscitation situations and their self-confidence in CPR skills.

## Methods

The survey took place between October and December in 2006 at three Norwegian hospitals: Buskerud Hospital Trust (Drammen), Ullevål University Hospital (Oslo) and the University Hospital of Northern Norway (Tromsø). These hospitals were offering three- and six-hours instructor-led compulsory Basic Life Support courses with or without AED to all hospital personnel. The courses were aimed at personnel outside critical care areas which excluded staff working in emergency rooms, intensive care and other critical care departments that offer Advanced Life Support courses for their personnel. The courses were run with a random mix of hospital staff groups through the year.

Data were collected by a questionnaire handed out to the course participants at course start without prior notice. The nurse who administered and collected the questionnaire informed the participants that ten minutes were given to complete the questionnaire before course start. The questionnaire was developed by the author and contained information about participation in the study being voluntarily and anonymous. The following demographic variables were registered: sex, age, years of work experience, profession (physician, registered nurse, enrolled nurse, midwife, biomedical laboratory scientist, radiographer/physiotherapist/occupational therapist, clerk personnel, or other) and place of work. The questionnaire inquired on the following variables: months since last CPR training, total numbers of CPR courses attended, number of resuscitation situations in any setting where respondents had taken an active part, and which of the following CPR actions they had performed during previous resuscitations (yes or no); chest compression, mouth-to-mouth or mask-to-mouth ventilation, attaching defibrillator, defibrillation, giving medication, and other efforts in previous or present CPR algorithms. The respondents were to give their own interpretation of their CPR skills by the following statement: "*I know how to perform CPR" *answered by a seven-point ordinal scale where 1 = not at all true and 7 = very true. Differences between means of reported self-confidence across gender, professional group, hospital and time since last CPR training (excluded those who had no previous training) were evaluated by ANOVA with the Bonferroni correction. Statistics were performed using STATA version 10.0 (StataCorp LP, Texas, USA). Approval to perform the study was obtained from all three hospitals. The study was part of an investigation of learning motivation among CPR course participants [6].

## Results

A total of 362 questionnaires were administered and 361 were completed. Most of the respondents were women (84%). Age varied between 20 and 71 years, with a median of 37 years. Length of work experience varied between the newly educated and up to 45 years, with a median of 7 years. The respondents were 194 registered nurses (53%), 57 enrolled nurses (16%), 29 biomedical laboratory scientists (8%), 20 clerk personnel (6%), 15 allied health personnel consisting of radiographers, physiotherapists and occupational therapists (4%), 13 midwives (4%), 8 physicians (2%) and 25 other personnel (7%). Reported places of work were medical-, surgical-, rehab-, psychiatric- and maternity departments, labs, x-ray departments, offices and outpatient clinics. Age and years of work experience were evenly distributed among the professional groups.

A total of 322 respondents (89.2%) had attended previous CPR training, 10.8% within the last six months. Table 1 presents the time interval since last CPR training by professions.

**Table 1:** Time interval since last cardiopulmonary resuscitation (CPR) training by staff group linked with confidence in skills (N = 361)

	Time since last CPR training						
Profession	≤ 6 months	7–12 months	13–24 months	> 24 months	No previous training	Not reported	Total
Reg. nurses	15.5 (30)	25.3 (49)	34.5 (67)	19.6 (38)	4.1 (8)	1.0 (2)	100.0 (194)
Enrolled nurses	5.3 (3)	17.5 (10)	35.1 (20)	22.8 (13)	7.0 (4)	12.3 (7)	100.0 (57)
Biomed. lab. sc.	0.0 (0)	10.3 (3)	27.6 (8)	13.8 (4)	44.8 (13)	3.5 (1)	100.0 (29)
Clerk pers.	5.0 (1)	10.0 (2)	15.0 (3)	35 (7)	30.0 (6)	10 (1)	100.0 (20)
Allied health pers.*	6.7 (1)	20 (3)	53.3 (8)	20 (3)	0.0 (0)	0.0 (0)	100.0 (15)
Midwives	0.0 (0)	7.7 (1)	0.0 (0)	53.9 (7)	23.1 (3)	15.3 (2)	100.0 (13)
Physicians	12.5 (1)	0.0 (0)	12.5 (1)	62.5 (5)	12.5 (1)	0.0 (0)	100.0 (8)
Others	12 (3)	8.0 (2)	24.0 (6)	32.0 (8)	16.0 (4)	12.0 (2)	100.0 (25)
Total	10.8 (39)	19.4 (70)	31.3 (113)	23.5 (85)	10.8 (39)	4.2 (15)	100.0 (361)

Self-confidence**	4.5	5.1	4.6	4.0	2.4	2.6	4.2

Values are percentages (n)

* Radiographers, physiotherapists and occupational therapists

**Values are means reported on a 7-point scale (see methods)

Numbers of CPR courses attended varied between one and 50, with a median of two courses. Time since last CPR training differed between one month and 30 years with a median of two years. Allied health personnel had the shortest time interval since last CPR training with median 1.3 years closely followed by registered nurses with median 1.4 years, while midwives and physicians had the longest time interval; median 3.4 and four years, respectively. Of those who had previous CPR training, amount of CPR courses attended was highest among registered nurses and physicians with median three courses and lowest among biomedical laboratory scientists who had attended median one course. The rest of the professions reported a median of two courses (data not shown).

A total of 118 respondents (32.7%) had taken an active part in a real resuscitation situation one or more times. Table 2 presents the distribution of used efforts in the CPR algorithm in the total sample by professions.

**Table 2:** Self reported use of cardiopulmonary resuscitation (CPR) skills in real cardiac arrest situations by staff group (N = 361)

	Efforts in the CPR algorithm						
Profession (n)	Compression	Ventilation	Attaching defibrillator	Defibrillation	Medication	Other efforts	No CPR experience
Reg. nurses (194)	27.8 (54)	21.1 (41)	5.7 (11)	3.1 (6)	10.8 (21)	12.9 (25)	61.9 (120)
Enrolled nurses (57)	31.6 (18)	19.3 (11)	1.8 (1)	0.0 (0)	0.0 (0)	8.8 (5)	64.9 (37)
Biomed. lab. sc. (29)	0.0 (0)	0.0 (0)	0.0 (0)	0.0 (0)	0.0 (0)	0.0 (0)	100.0 (29)
Clerk pers. (20)	15.0 (3)	15.0 (3)	0.0 (0)	0.0 (0)	0.0 (0)	0.0 (0)	85.0 (17)
Allied health pers.* (15)	20.0 (3)	20.0 (3)	0.0 (0)	0.0 (0)	0.0 (0)	0.0 (0)	80.0 (12)
Midwives (13)	38.5 (5)	38.5 (5)	7.7 (1)	0.0 (0)	7.7 (1)	0.0 (0)	53.9 (7)
Physicians (8)	87.5 (7)	87.5 (7)	37.5 (3)	37.5 (3)	50.0 (4)	50.0 (4)	12.5 (1)
Others (25)	16.0 (4)	12.0 (3)	0.0 (0)	0.0 (0)	0.0 (0)	8.0 (2)	80.0 (20)
All hospital staff (361)	26.0 (94)	20.2 (73)	4.4 (16)	2.5 (9)	7.2 (26)	10.0 (36)	67.3 (243)

Values are percentages (n)

* Radiographers, physiotherapists and occupational therapists

Number of times the respondents had acted in resuscitation varied between one and 200 with a median of two cardiac arrests, and a total of 14% had acted in more than one resuscitation situation. Among those who had acted in resuscitation physicians and the group of other personnel reported on a median of three cardiac arrests, registered nurses, midwives and allied health personnel reported on a median of two cardiac arrests while enrolled nurses and clerk personnel reported on acting at a median of one cardiac arrest (data not shown).

For all respondents the mean value for the statement "*I know how to perform CPR" *was 4.5. Mean scores are presented together with time interval since last CPR training in table 1. The score was only significantly different for respondents who reported a time interval of more than two years since last training. The score was evenly distributed in all personnel groups and showed no gender difference (data not shown). Overall, none of the answers differed among the hospitals.

## Discussion

Most of the hospital staff reported on previous CPR training, but only one of ten had participated in CPR training within the last six months as recommended by national guidelines. One of three of all hospital personnel reported taken active part in CPR in a real resuscitation situation, but few had acted in more than one resuscitation situation. One of four had performed chest compressions, one of five had performed mouth-to-mouth or mask-to-mouth ventilations, one of 20 had attached a defibrillator and half of these had performed defibrillation. In comparison a similar British study performed at three hospitals with several years of ongoing CPR training programmes found that almost one third of all hospital staff had participated in CPR training within the last six months [7]. They found that almost half of all hospital personnel had attended a cardiac arrest and that chest compressions and bag-valve-mask use were among the most reported skills used in arrests [7]. The discrepancy between training frequency and real resuscitation experience is larger in our study, but the study design makes it impossible to investigate whether it is the lack of training that keeps some groups of personnel from acting in resuscitation. Reports from different hospitals show internationally similar incidence- and survival rates [1,2]. As respondents in this study only reported on their own resuscitation experiences, no information is provided on arrests where they had not taken active part in CPR, nor is it made comparisons with the respective hospitals cardiac arrest incidence.

Registered nurses reported highest and physicians lowest compliance to recommended training frequency, and over 40% of the biomedical laboratory scientists and 30% of the clerk personnel reported no previous training. More surprisingly, some respondents among both physicians and nursing staff (registered nurses, enrolled nurses and midwives) reported no participation in any CPR training before. A Finnish study of CPR use and training at 55 hospitals found that regular CPR training was more common among nurses than among physicians [8]. Also defibrillation training was more common among nurses, but defibrillation was mostly performed by physicians on general wards [8], which can partly be explained by the traditional role of the physician as being in charge of the medical treatment. Physicians and nursing staff had the highest participation in cardiac arrests, which is consistent with previous findings [7]. Among nursing staff, the group of midwives reported on the largest mean time interval since last training, had the largest proportion of respondents without previous CPR training, and had the highest participation in resuscitation. The high participation in resuscitation among midwives may partly be explained by former work experience in general nursing, as Norwegian midwives have basic education as registered nurses, but the midwives had the same mean age as the registered nurses in this survey. It may also be due to experience with neonatal resuscitation as the questionnaire did not distinguish between adult, pediatric and neonatal resuscitation attempts.

All hospital personnel reported moderate self-confidence in CPR skills, which only decreased when time since last training was more than two years. Similar results have been reported [9]. Even those who reported no previous CPR training had a mean value over one, which can be due to knowledge of resuscitation without actually having trained on the practical skills. Self-confidence has been associated with skills in a recent study [10], though earlier findings have shown no such association [11]. Confidence with no association to time since training may be a false trust in previous learned skills or individuals may have given expected answers. As the sample was conducted from convenience and some of the staff groups were small, caution should be present when interpreting the results. Another limitation is that self reported CPR training and skills usage was not restricted to the in-hospital setting.

The importance of immediate start of CPR and early defibrillation has led to large time-consuming and costly training programmes and distribution of AEDs in most hospital settings. It is well-known that CPR skills degrade quickly after training [1,4,12] It is questionable whether the fulfilment of the recommended training guidelines is realistic in all hospital settings. Training should be targeted at skills most likely to be used in the clinical setting [7]. A study of experienced and specially trained physicians in an acute care setting showed satisfactory CPR performance in cardiac arrests [13]. The authors concluded that this was due to frequent training and frequent attendance at cardiac arrests in their work setting [13]. A follow up of nurses participating at an Immediate Life Support course showed that only a very small proportion had used the advanced skills learned in the course when attending a cardiac arrest [14]. It was concluded that training alone was insufficient to increase use of more advanced skills and that interval between course attendance and first cardiac arrest was of importance to whether the skills were used in a cardiac arrest situation [14]. It may seem like regular training *and *exposure to cardiac arrests are of importance to retain skills. Skogvoll and colleagues reported an incidence of CPR attempts of 54.2 per 1000 beds per year outside critical care areas in a 900-bed Norwegian university hospital, and for all in-hospital deaths CPR was attempted in 5% of the cases [3]. This incidence is too low to expose most in-hospital staff. A Finnish study showed that after a remodelling of hospital resuscitation management the survival of cardiac arrest outside critical care areas improved, but the use of basic life support before arrival of the cardiac arrest team was not significantly changed [15]. Cost-effectiveness of CPR training and use has been highlighted before [7] and it may be that it is still too early to see the effects of organizational changes like greater emphasis on in-hospital CPR training.

## Conclusion

A minority of those who took part in this study had participated in resuscitation situations, though most of them had CPR training. Time interval since last CPR training were beyond the recommended guidelines of six months among almost 90% of the respondents, but confidence in CPR skills were unchanged until this time interval exceeded two years. This small self-report study indicates two discrepancies; the gap between recommendations from recommended guidelines and reality in CPR training, and the gap between training frequency and real resuscitation experience.

## Competing interests

The author declares that they have no competing interests.
